# Perceived social support and life engagement among older adults using thermal health tourism services: the moderating role of aging anxiety

**DOI:** 10.3389/frhs.2026.1901204

**Published:** 2026-09-23

**Authors:** Kadriye Sönmez

**Affiliations:** Medical Documentation and Secretarial Programme, Plato Vocational School, Istanbul Topkapi University, Istanbul, Türkiye

**Keywords:** aging anxiety, life engagement, moderated regression analysis, older adults, perceived social support, thermal health tourism

## Abstract

**Background:**

Life engagement is a central dimension of psychological functioning in later life. Its association with perceived social support varies across levels of aging-related concern, and evidence from thermal health tourism settings remains limited.

**Objective:**

This study examined the associations of perceived social support and aging anxiety with life engagement among older adults using thermal health tourism services, including the interaction between perceived social support and aging anxiety. Source-specific support analyses described the relative contributions of significant-other, family, and friend support.

**Methods:**

This cross-sectional study used 415 deduplicated questionnaires from adults aged 65 years and older at one thermal health tourism facility. Scale scores required at least 80% item completion. Pearson correlations and ordinary least squares regression with HC3 heteroskedasticity-consistent standard errors formed the primary analysis. Robust regression, median quantile regression, bootstrap estimation, strict complete-item scoring, source-specific models, dominance analysis, and commonality analysis provided sensitivity and secondary results. The primary adjusted model included 384 complete cases.

**Results:**

Perceived social support was positively associated with life engagement [b = 0.106, 95% CI (0.048, 0.164), *p* < .001], and aging anxiety was negatively associated with life engagement [b = −0.326, 95% CI (−0.474, −0.179), *p* < .001]. The interaction was negative [b = −0.061, 95% CI (−0.118, −0.005), *p* = .034], with an incremental R^2^ of.014. The positive support-life engagement association was statistically significant below an aging-anxiety score of 3.15. The interaction confidence interval included zero in the strict complete-item and median quantile sensitivity analyses. The three support sources were highly correlated, and their combined incremental R^2^ was.028 in the source-specific analysis.

**Conclusion:**

Among voluntary users of one thermal health tourism facility, higher perceived social support and lower aging anxiety were associated with higher life engagement. The support-life engagement association was smaller at higher aging-anxiety levels. The cross-sectional interaction was modest and sensitive to the missing-item rule and conditional-distribution model.

## Introduction

Population aging is a central public health and social policy issue. The World Health Organization (1) estimates that the number of people aged 60 years and older will rise from 1.1 billion in 2023 to 1.4 billion by 2030, and the United Nations projects continued growth in the oldest-old population (2). Later-life health encompasses mental health, social connectedness, everyday functioning, and longevity (3, 4). Life engagement and social connection therefore represent central dimensions of healthy aging.

Perceived social support is consistently associated with later-life well-being. Stronger social support is associated with better quality of life (5), and loneliness partly accounts for the association between support and physical and mental outcomes (6). Institutional research also links social support with psychological pathways involved in successful aging (7). Support from family, friends, and close others occupies distinct positions within an older adult's social network (8). Living arrangements, widowhood, retirement, and health limitations shape the availability and salience of these ties.

Life engagement reflects meaningful goals, sustained involvement, and the value assigned to daily activities (9, 10). Purpose-related functioning is associated with psychological well-being and health-promoting behavior in older age (11, 12). Longitudinal evidence links favorable social-network types with stronger purpose in life (8), and social participation is associated with mental well-being through purpose in life (13). These findings place social context within the empirical study of later-life engagement.

Aging-related perceptions form the psychological context in which social resources are experienced. Positive self-perceptions of aging are associated with quality of life (4) and psychological well-being (14), while anxiety symptoms are common and underdetected in older populations (3, 15). Interventions directed at self-perceptions of aging have produced improvements in psychosocial outcomes (16), and socioeconomic position is associated with aging anxiety through stressors and aging perceptions (17). These findings support a resource-threat framework in which perceived support represents a relational resource, aging anxiety represents an aging-related threat appraisal, and life engagement represents meaningful involvement in life.

Thermal and wellness travel provides a health-seeking service context for studying these psychosocial associations. Tourism research links later-life travel with healthy aging, social participation, and age-friendly service design (18). Spa motivations are associated with post-trip life satisfaction among older visitors (19), and wellness tourism experiences are associated with restorative appraisals and well-being (20). Existing studies emphasize motivations, segmentation, life satisfaction, and broad well-being outcomes. Source-specific support, aging anxiety, and life engagement remain less developed within this recruitment context.

The study addressed three empirical gaps: the association between perceived social support and life engagement among older thermal-service users; the interaction between perceived support and aging anxiety; and the overlapping contributions of significant-other, family, and friend support, which have distinct associations with later-life adaptation and purpose (6, 8).

The resource-threat framework generated three hypotheses. Higher perceived social support was expected to be associated with higher life engagement (H1), higher aging anxiety was expected to be associated with lower life engagement (H2), and the positive association between perceived social support and life engagement was expected to be smaller at higher levels of aging anxiety (H3). Source-specific support analyses were designated secondary because the MSPSS subscales represent correlated components of the same support system. The thermal facility served as the recruitment setting; facility characteristics were not modeled as explanatory exposures.

## Methods

### Study design and setting

This cross-sectional, questionnaire-based observational study followed the Strengthening the Reporting of Observational Studies in Epidemiology recommendations (21). Participants were recruited at a single thermal health tourism facility in Türkiye during the approved field period. The facility provided thermal health and wellness services to older adults and served solely as the recruitment setting for the study. The facility name and more specific geographical information are not reported to preserve institutional confidentiality. No facility-level exposure was included in the analysis. All observations came from one site; therefore, facility codes, facility fixed effects, and cluster-robust standard errors were not applicable.

### Participants and procedure

A facility-based convenience sample included service users aged 65 years or older who understood the study information, provided digital informed consent, and completed the electronic questionnaire in Turkish. Age eligibility was self-reported at enrollment. No formal cognitive screening instrument was administered. The exact age category was unavailable for 17 enrolled participants and was retained as missing in the analytic file. The source dataset contained submitted questionnaires; counts of people approached, declining, or leaving the form before submission were not recorded, so an approach-based participation rate was unavailable.

The standardized electronic questionnaire contained a sociodemographic form and three psychometric instruments. Study information and digital consent preceded the survey items. Participation was voluntary, responses were confidential, and discontinuation carried no penalty. The electronic form applied the same item wording, response options, and order to all respondents. The analytic dataset contained no interviewer identifier or directly identifying participant field.

### Measures

#### Perceived social support

Perceived social support was assessed with the 12-item Multidimensional Scale of Perceived Social Support (MSPSS) (22) and its Turkish version (23). Items use a 7-point response scale and form significant-other, family, and friend subscales. The total score was the mean of available items when at least 10 of 12 items were answered. Each four-item subscale required all four responses. Higher scores indicated greater perceived social support.

#### Life engagement

Life engagement was measured with the six-item Life Engagement Test (24) and its Turkish adaptation (25). Responses ranged from 1 (strongly disagree) to 5 (strongly agree). Items 1, 3, and 5 were reverse-scored. The life engagement score was the mean of available items when at least five of six items were answered. Higher scores indicated greater life engagement.

#### Relational aging anxiety

Aging-related concerns were measured with the 16-item Turkish Relational Aging Anxiety Scale (26), developed from the relational ageism framework (27). Responses ranged from 1 to 5. Items 3, 8, 11, and 13 were retained in the field direction; Items 1, 2, 4, 5, 6, 7, 9, 10, 12, 14, 15, and 16 were reverse-scored so that higher values indicated greater aging anxiety. The total score was the mean of available items when at least 13 of 16 items were answered. The total score was treated as an operational composite; subdimension scores were excluded from hypothesis testing.

#### Sociodemographic and study-specific variables

Covariates were age group (65–74, 75–84, ≥85 years), sex, educational level, thermal-facility visit frequency, primary visit purpose, chronic disease status, living arrangement, and social insurance status. All covariates were categorical. Reference categories were 65–74 years, men, university/postgraduate education, one visit every several years, physical treatment, chronic disease present, living with a spouse, and social insurance present. The model contained 17 covariate indicator variables.

### Data management and statistical analysis

The source file contained 425 submitted questionnaires. Data screening covered response ranges, missing values, response structure, and exact duplicate patterns. Duplicate detection used all 42 non-timestamp fields because participant identifiers were absent. Ten later records matched an earlier record on every non-timestamp field; the first record in each exact-match sequence was retained, yielding 415 unique analytic records. Scale means were computed with the prespecified 80% item-completion rules. Primary regression used complete cases across the outcome, focal predictors, and covariates. A strict complete-item sensitivity analysis required all MSPSS, LET, and aging-anxiety items. A second sensitivity analysis retained the 10 exact-match records.

Cronbach's alpha was calculated among respondents with complete item data for each scale. Means and standard deviations used all scores meeting the 80% rule. Categorical variables were summarized with frequencies, percentages, and explicit missing counts. Pearson correlations used pairwise available scores, with the exact pairwise denominator reported in each table cell. Primary-model complete cases were compared with excluded records on available scale scores using Welch tests and on categorical variables using chi-square tests.

The primary model regressed life engagement on mean-centered perceived social support, mean-centered aging anxiety, their product term, and the prespecified covariates. Ordinary least squares coefficients were reported as unstandardized b values with HC3 heteroskedasticity-consistent standard errors (28). Mean-centering preceded construction of the interaction term (29). The model included 20 predictors plus the intercept. Conditional support slopes were estimated at the aging-anxiety mean and at one standard deviation below and above the mean. The Johnson-Neyman procedure identified the aging-anxiety values for which the adjusted support slope differed from zero. A sensitivity calculation based on G*Power conventions (30) showed that *n* = 384, *α* = .05, one tested interaction term, and 20 total predictors corresponded to 80% power for f^2^ = .021.

Sensitivity analyses comprised Huber M-estimation, median quantile regression, strict complete-item scoring, and duplicate-retained estimation. Participant-level nonparametric bootstrap resampling used 2,000 samples, a percentile confidence interval, and random seed 20260731 for the interaction term (31). A formal three-degree-of-freedom support-by-living-arrangement interaction tested slope heterogeneity. Secondary source-specific models entered each MSPSS subscale separately and then simultaneously, with aging anxiety and the covariates retained. General dominance weights averaged incremental R^2^ across every subset of the three support sources. Commonality coefficients decomposed the source-attributable incremental R^2^ into unique, pairwise shared, and three-way shared components. Dominance and commonality intervals used 500 participant-level bootstrap samples with seed 20260731. All tests were two-sided with *α* = .05.

### Ethical considerations

The institutional ethics committee approved the study before participant recruitment, and the participating thermal facility authorized data collection. Digital informed consent was obtained before questionnaire access. Participation was voluntary, withdrawal carried no penalty, and the analytic dataset contained no direct identifiers. The ethics approval identifiers and approved field dates were entered consistently in the submission record and study documentation.

## Results

The source file contained 425 questionnaires. Exact-pattern screening removed 10 later duplicate records and retained 415 unique records. Table 1 presents the sample characteristics and missing counts. The primary scale-score denominators were 408 for MSPSS total, 410 for life engagement, and 409 for aging anxiety. Thirty-one records lacked at least one variable required by the adjusted model, producing a primary-model sample of 384.

**Table 1:** Sample characteristics after duplicate removal.

Variable	Category	n	%
Age group	65–74	184	44.3
	75–84	132	31.8
	85 and older	82	19.8
	Missing	17	4.1
Sex	Women	241	58.1
	Men	174	41.9
Educational level	University/postgraduate	174	41.9
	Primary/middle school	131	31.6
	Not literate	55	13.3
	High school	54	13.0
	Missing	1	0.2
Thermal-visit frequency	Once every several years	125	30.1
	Once a year	106	25.5
	First-time visitor	96	23.1
	Several times a year	83	20.0
	Missing	5	1.2
Primary visit purpose	Physical treatment (e.g., joint or muscle pain)	180	43.4
	Rest and stress relief	106	25.5
	Socializing and meeting new people	71	17.1
	Spending time with family	53	12.8
	Missing	5	1.2
Chronic disease status	Yes	292	70.4
	No	121	29.2
	Missing	2	0.5
Living arrangement	With spouse	149	35.9
	With children	125	30.1
	Alone	108	26.0
	Nursing home	24	5.8
	Missing	9	2.2
Social insurance	Yes	337	81.2
	No	73	17.6
	Missing	5	1.2

Note. Percentages use the deduplicated sample (*N* = 415); missing responses are displayed for every variable with missing data. Sex had no missing values.

Table 2 presents score distributions and internal consistency. Cronbach's alpha was.978 for MSPSS total,.819 for life engagement, and.929 for aging anxiety. Mean scores were 4.62 (SD = 1.98) for MSPSS total, 3.67 (SD = 0.89) for life engagement, and 2.49 (SD = 0.87) for aging anxiety. The MSPSS source subscales also showed high internal consistency (*α* = .967-.971).

**Table 2:** Internal consistency and descriptive statistics for study measures.

Measure	Scale range	*α* (n)	Score n	M	SD
MSPSS total	1–7	0.98 (379)	408	4.62	1.98
MSPSS significant other	1–7	0.97 (396)	396	4.43	2.25
MSPSS family	1–7	0.97 (396)	396	4.87	2.09
MSPSS friends	1–7	0.97 (402)	402	4.59	1.96
Life engagement	1–5	0.82 (400)	410	3.67	0.89
Aging anxiety total	1–5	0.93 (380)	409	2.49	0.87

α was calculated from complete item sets; α denominators are shown in parentheses. Score *n* reflects the prespecified item-completion rule. MSPSS, Multidimensional Scale of Perceived Social Support.

Table 3 and Figure 1 present the pairwise correlations. MSPSS total was positively correlated with life engagement (r = .440) and negatively correlated with aging anxiety (r = -.477). Aging anxiety was negatively correlated with life engagement (r = -.411). Family support had the largest bivariate correlation with life engagement (r = .452), followed by friend support (r = .407) and significant-other support (r = .380). Correlations among the three support sources ranged from.808 to.861.

**Table 3:** Pearson correlations among perceived social support, life engagement, and total aging anxiety.

Variable	1	2	3	4	5	6
1. MSPSS total	—					
2. Significant other	.934 (396)	—				
3. Family	.946 (396)	.811 (384)	—			
4. Friends	.943 (402)	.808 (390)	.861 (391)	—		
5. Life engagement	.440 (408)	.380 (396)	.452 (396)	.407 (402)	—	
6. Aging anxiety	-.477 (407)	-.391 (396)	-.499 (395)	-.464 (401)	-.411 (409)	—

Lower-triangle cells report Pearson r with the pairwise available-case *n* in parentheses. Variable labels: 1 = MSPSS total; 2 = significant-other support; 3 = family support; 4 = friend support; 5 = life engagement; 6 = aging anxiety.

**Figure 1:**
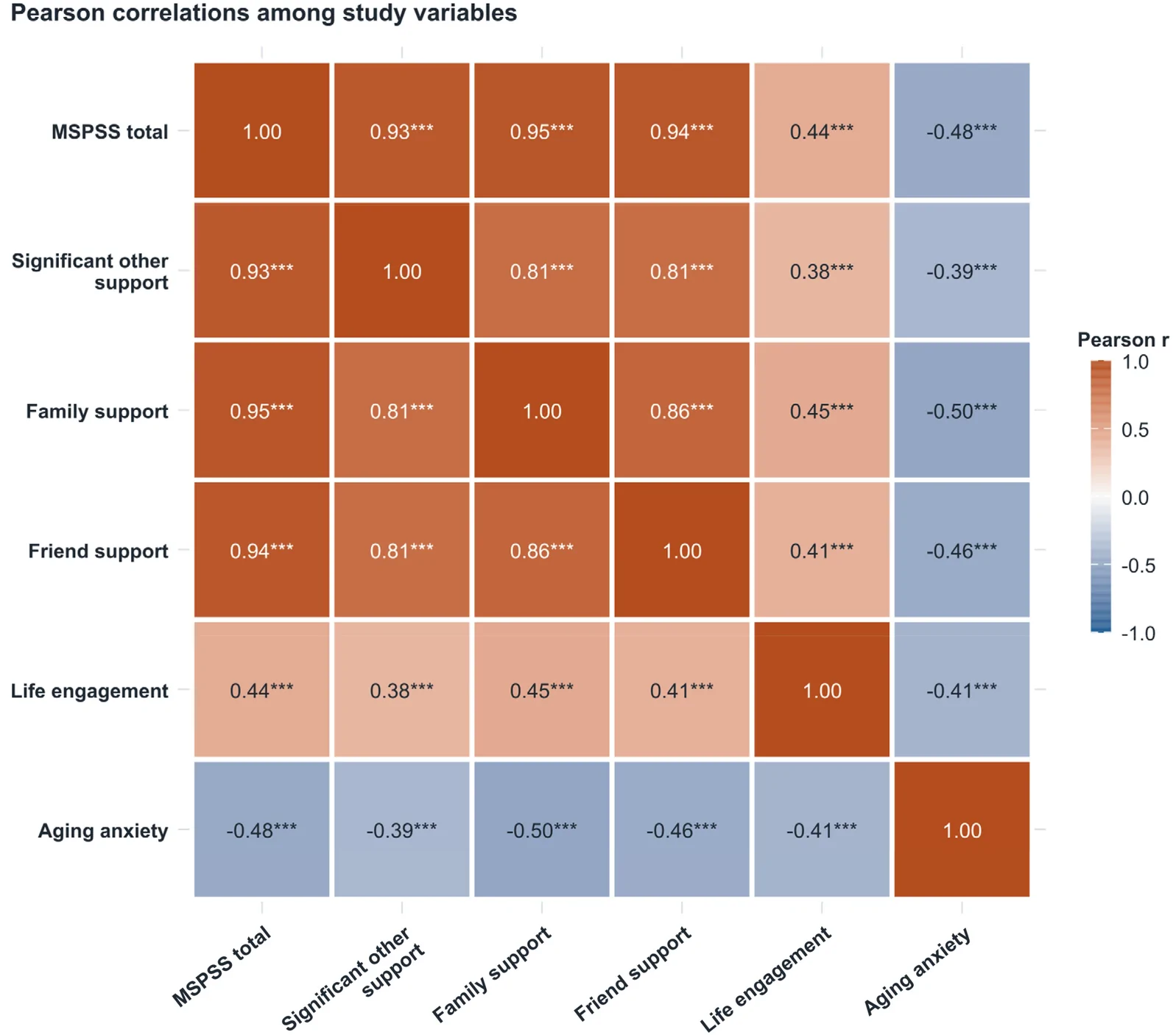
Pearson correlation matrix of perceived social support, life engagement, and aging anxiety.

The adjusted model was statistically significant, robust F(20, 363) = 12.72, *p* < .001, with R^2^ = .349 and adjusted R^2^ = .313 (Table 4). At the mean aging-anxiety score, perceived social support was positively associated with life engagement [b = 0.106, 95% CI (0.048, 0.164), *p* < .001]. At the mean support score, aging anxiety was negatively associated with life engagement [b = −0.326, 95% CI (−0.474, −0.179), *p* < .001]. The interaction was negative [b = −0.061, 95% CI (−0.118, −0.005), *p* = .034] and added *Δ*R^2^ = .014.

**Table 4:** Fully adjusted regression models for life engagement.

Predictor	b	HC3 SE	95% CI	t	p
Constant	3.784	.138	[3.513, 4.055]	27.453	<.001
Perceived social support (centered)	.106	.029	[.048,.164]	3.612	<.001
Aging anxiety (centered)	-.326	.075	[-.474, -.179]	−4.344	<.001
Social support × aging anxiety	-.061	.029	[-.118, -.005]	−2.123	.034
Age 75–84 (ref: 65–74)	-.352	.097	[-.543, -.161]	−3.633	<.001
Age ≥85 (ref: 65–74)	-.447	.107	[-.658, -.236]	−4.168	<.001
Women (ref: men)	-.113	.084	[-.278,.053]	−1.338	.182
High school (ref: university/postgraduate)	.101	.124	[-.143,.346]	.814	.416
Not literate (ref: university/postgraduate)	.188	.141	[-.089,.465]	1.335	.183
Primary/middle school (ref: university/postgraduate)	-.005	.100	[-.202,.191]	-.052	.958
Once a year (ref: once every several years)	.082	.106	[-.127,.291]	.772	.441
Several times a year (ref: once every several years)	.010	.104	[-.195,.215]	.097	.923
First visit (ref: once every several years)	.175	.110	[-.041,.391]	1.595	.112
Time with family (ref: physical treatment)	.086	.127	[-.163,.334]	.677	.499
Rest/stress relief (ref: physical treatment)	.050	.108	[-.163,.262]	.459	.646
Socializing (ref: physical treatment)	.154	.122	[-.086,.394]	1.262	.208
No chronic disease (ref: yes)	-.011	.100	[-.207,.185]	-.112	.911
Nursing home (ref: spouse)	-.606	.193	[-.986, -.226]	−3.136	.002
Alone (ref: spouse)	-.005	.105	[-.212,.202]	-.044	.965
With children (ref: spouse)	.046	.094	[-.139,.231]	.485	.628
No social insurance (ref: yes)	-.193	.102	[-.393,.007]	−1.903	.058

Coefficients are unstandardized b values from the primary ordinary least squares model with HC3 standard errors (*n* = 384). Perceived social support and aging anxiety were mean-centered. Reference categories are shown in the predictor labels. Model R^2^ = .349; adjusted R^2^ = .313; interaction *Δ*R^2^ = .014; robust F(20, 363) = 12.72, *p* < .001.

The conditional support slope was 0.160 [95% CI (0.079, 0.241), *p* < .001] at one standard deviation below the aging-anxiety mean, 0.106 [95% CI (0.048, 0.164), *p* < .001] at the mean, and 0.052 [95% CI (−0.020, 0.124), *p* = .154] at one standard deviation above the mean. The Johnson-Neyman boundary was 3.15 on the observed 1–5 aging-anxiety scale; the positive adjusted support slope was statistically significant below this value (Figure 2).

**Figure 2:**
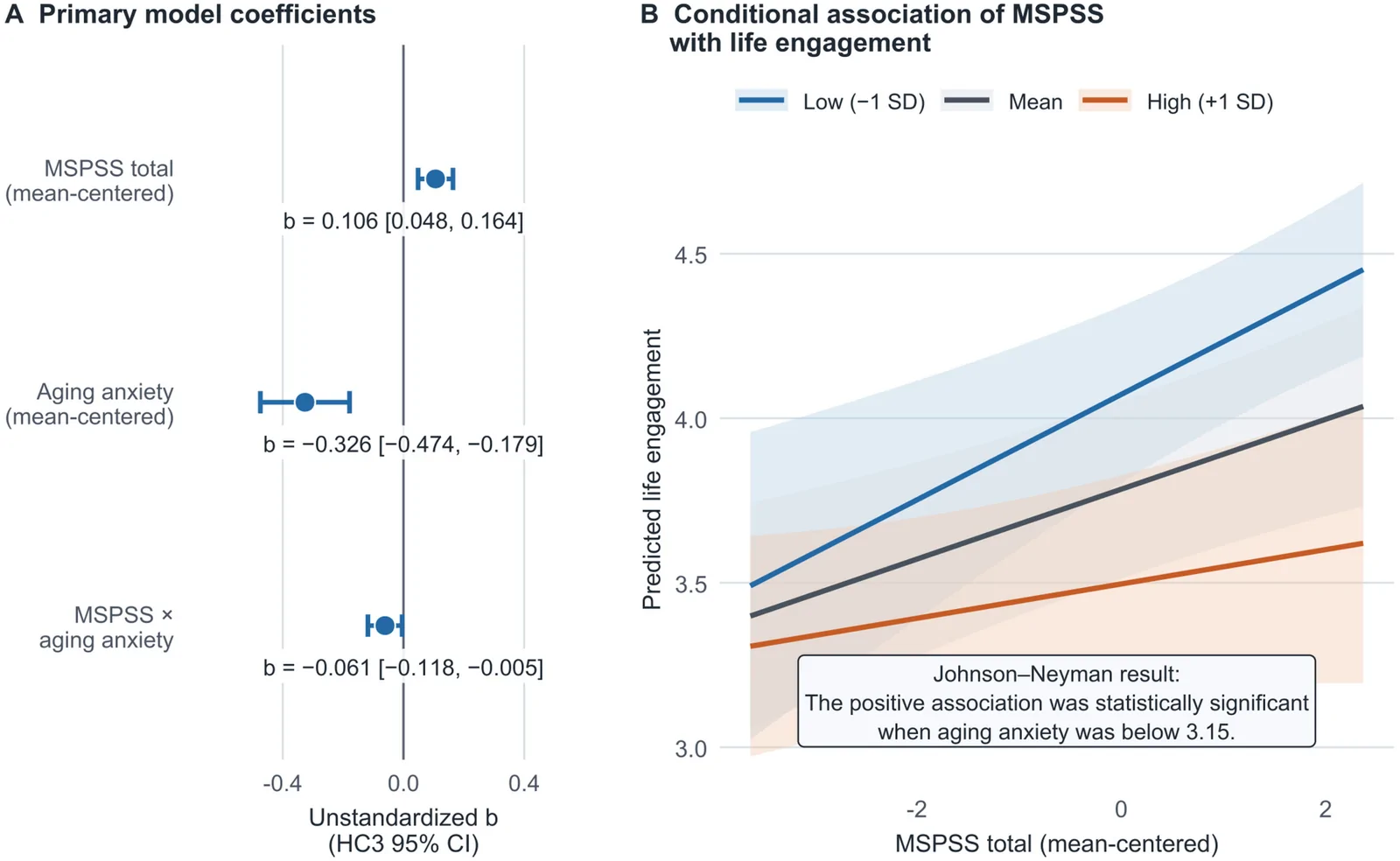
Adjusted associations and moderation of the perceived social support-life engagement association. **(A)** Unstandardized regression coefficients and HC3 95% confidence intervals for perceived social support, aging anxiety, and their interaction in the primary adjusted model. **(B)** Conditional association between perceived social support and predicted life engagement at low (−1 SD), mean, and high (+1 SD) levels of aging anxiety. The Johnson-Neyman analysis indicated that the positive adjusted association was statistically significant at aging-anxiety scores below 3.15.

**Figure 3:**
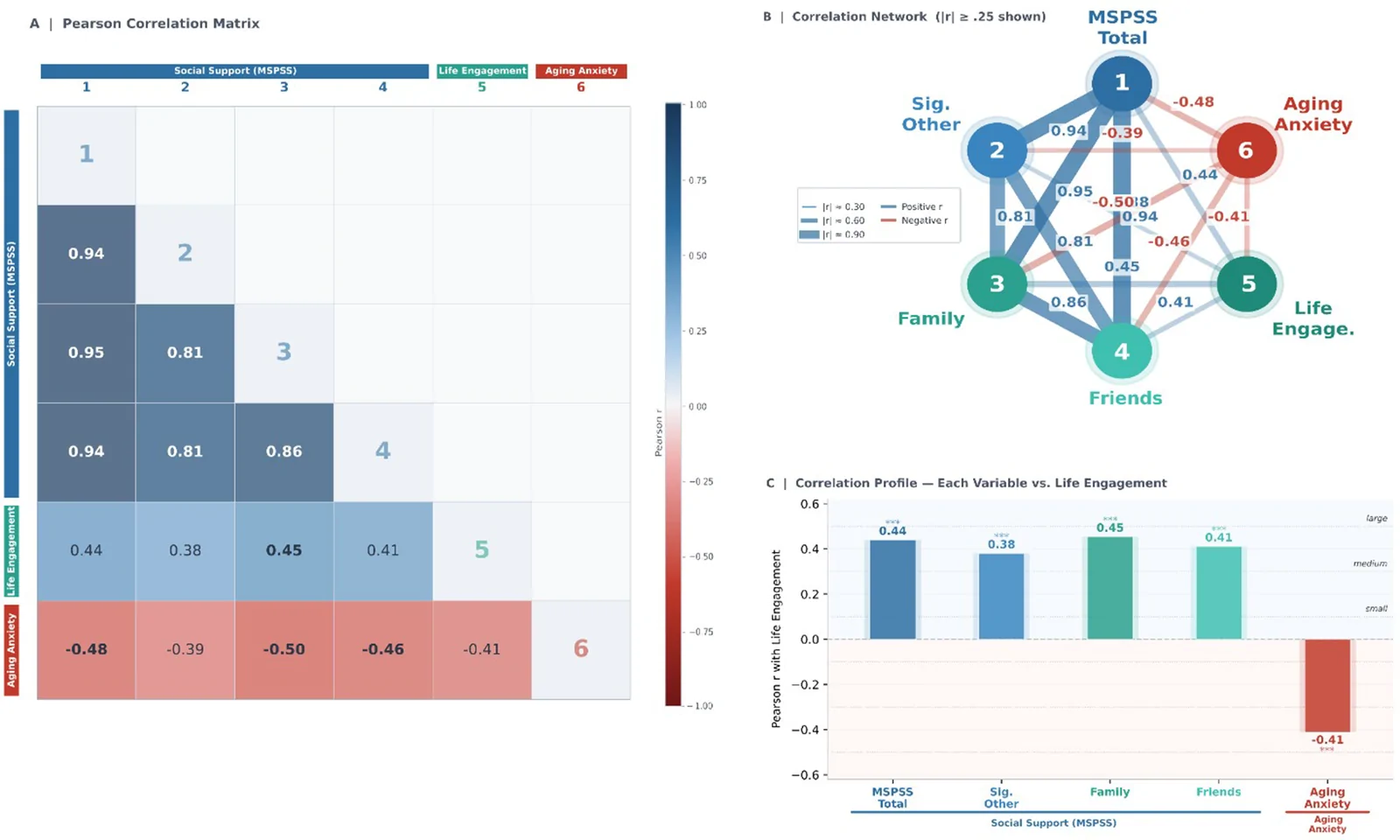
Correlation structure among perceived social support, life engagement, and aging anxiety. (A) Pearson correlation matrix showing pairwise associations among MSPSS total, significant-other support, family support, friend support, life engagement, and aging anxiety. (B) Correlation network illustrating the direction and magnitude of pairwise associations among the study variables. (C) Correlation profile showing the bivariate associations of MSPSS total, significant-other support, family support, friend support, and aging anxiety with life engagement.

The interaction estimate remained negative in Huber M-estimation [b = −0.051, 95% CI (−0.089, −0.013), *p* = .008] and the 2,000-sample participant bootstrap [mean b = −0.062, percentile 95% CI (−0.115, −0.012)]. Its confidence interval included zero in median quantile regression [b = −0.042, 95% CI (−0.086, 0.003), *p* = .069] and in the strict complete-item analysis [*n* = 334; b = −0.057, 95% CI (−0.120, 0.007), *p* = .081]. Retaining exact-match submissions produced a similar estimate [*n* = 392; b = −0.058, 95% CI (−0.114, −0.003), *p* = .039].

The Breusch-Pagan test detected heteroskedasticity, LM(20) = 61.42, *p* < .001, supporting HC3 inference. Ramsey RESET showed no evidence of functional-form misspecification, F(1, 362) = 0.13, *p* = .722. The maximum variance inflation factor was 1.70, maximum Cook's distance was.034, and maximum leverage was.113. A formal support-by-living-arrangement interaction was not significant, robust F(3, 360) = 1.44, *p* = .230. Available focal scale means were similar between included and excluded records (all *p* ≥ .136); categorical distributions differed for sex, education, chronic disease, and living arrangement (all *p* ≤ .016).

The source-specific analysis used 358 records with complete four-item MSPSS subscales and complete model variables (Table 5). Source correlations ranged from.808 to.861, and source-model variance inflation factors ranged from 3.69 to 5.24. Each source was positively associated with life engagement in separate adjusted models: significant-other support b = 0.070, family support b = 0.093, and friend support b = 0.090. In the simultaneous model, the three source coefficients had confidence intervals containing zero. The three sources increased R^2^ from.318 to.346 (incremental R^2^ = .028). General dominance weights allocated 41.2% of this increment to family support, 35.0% to friend support, and 23.8% to significant-other support; the bootstrap intervals overlapped.

**Table 5:** Support-source overlap, separate adjusted models, and dominance analysis.

Component	Estimate	95% CI/companion	p/%	Rank/model
**Panel A. Support-source overlap**				
Significant other vs. family	.839	Pairwise Pearson r		
Significant other vs. friends	.807	Pairwise Pearson r		
Family vs. friends	.869	Pairwise Pearson r		
Significant other support	VIF 3.69	Tolerance 0.271		
Family support	VIF 5.24	Tolerance 0.191		
Friend support	VIF 4.45	Tolerance 0.225		
**Panel B. Adjusted source-specific models**				
Significant other support (separate)	.070	[.019,.120]	.007	R^2^ 0.337
Family support (separate)	.093	[.036,.151]	.002	R^2^ 0.344
Friend support (separate)	.090	[.030,.149]	.003	R^2^ 0.342
Significant other support (mutually adjusted)	.008	[-.070,.086]	.841	R^2^ 0.346
Family support (mutually adjusted)	.056	[-.040,.152]	.251	R^2^ 0.346
Friend support (mutually adjusted)	.038	[-.056,.132]	.428	R^2^ 0.346
**Panel C. General dominance analysis**				
Family support	.0116	[.0025,.0313]	41.2%	1
Friend support	.0098	[.0020,.0317]	35.0%	2
Significant other support	.0067	[.0013,.0229]	23.8%	3

Source models included aging anxiety and all primary-model covariates (*n* = 358). VIF, variance inflation factor. General dominance weights sum to the source-attributable incremental R^2^ (.028); 95% intervals use 500 participant-level bootstrap samples.

Commonality analysis allocated 61.6% of the source-attributable incremental R^2^ to variance shared across all three support sources (Table 6). Unique components were 0.0001 for significant-other support, 0.0027 for family support, and 0.0014 for friend support. Pairwise shared components were positive and small. The decomposition shows that most source-related explanatory variance was common to the three MSPSS subscales.

**Table 6:** Commonality analysis of MSPSS support-source subscales in relation to life engagement.

Component	Coefficient	95% bootstrap CI	Share of incremental R^2^ (%)
Unique: Significant other support	.0001	[.0000,.0126]	0.3
Unique: Family support	.0027	[.0000,.0157]	9.7
Unique: Friend support	.0014	[.0000,.0152]	5.0
Shared: Significant other support + Family support	.0012	[-.0040,.0084]	4.4
Shared: Significant other support + Friend support	.0004	[-.0028,.0041]	1.4
Shared: Family support + Friend support	.0049	[-.0021,.0201]	17.6
Shared: Significant other support + Family support + Friend support	.0173	[.0005,.0424]	61.6

The baseline model contained aging anxiety and all primary-model covariates. Baseline R^2^ = .318, full-model R^2^ = .346, source-attributable incremental R^2^ = .028, *n* = 358. Confidence intervals use 500 participant-level bootstrap samples. Components sum to the incremental R^2^; negative values, when present in a bootstrap sample, represent suppressor-related overlap.

## Discussion

Three findings summarize the analysis. Perceived social support was positively associated with life engagement, aging anxiety was negatively associated with life engagement, and the adjusted support-life engagement association was smaller at higher aging-anxiety values. The interaction explained 1.4% additional variance and was statistically significant in the HC3 model, Huber model, and participant bootstrap. The median quantile and strict complete-item confidence intervals included zero. Source-specific results were dominated by variance shared across significant-other, family, and friend support.

The positive support-life engagement association is consistent with longitudinal evidence linking meaning in life with health, emotional well-being, and daily functioning (32). Active social engagement is associated with health-related quality of life (33), and regular social interaction is associated with affective well-being (34). Nursing-home research also links social support with successful aging through meaning in life (7). The present cross-sectional association extends this pattern to older adults voluntarily using a thermal health tourism service.

The negative interaction is consistent with a resource-threat framework. Research in older adults with chronic illness has documented joint associations among social support, psychological well-being, and burdensomeness (35). Intergenerational support also shows distinct emotional and practical associations with mental health (36). In the present sample, the adjusted support slope declined from 0.160 at low aging anxiety to 0.052 at high aging anxiety, and statistical significance ended at an aging-anxiety score of 3.15. This pattern describes conditional association within the observed sample.

The interaction magnitude was modest. Aging-related negative cognitions are associated with poorer psychological well-being (37), and psychological interventions in older populations are associated with improvements in emotional symptoms and quality of life (38). The current data identify aging anxiety as a variable that accompanies heterogeneity in the support-life engagement association. Longitudinal and intervention studies are required to estimate direction and clinical relevance.

The source-specific coefficients reflect substantial shared variance. Collinearity increases coefficient uncertainty when correlated predictors enter the same model (39). The MSPSS source correlations ranged from.808 to.861, and simultaneous source coefficients had wide intervals containing zero. Separate adjusted models produced positive coefficients for every source, consistent with prior source-specific research (40). These estimates describe correlated dimensions of perceived support and provide no basis for an isolated causal effect of one source.

Dominance analysis compares relative predictor contributions across subset models (41), and variance-decomposition methods quantify shared explanatory variance (42, 43). Family support had the largest point estimate for the general dominance weight, followed by friend and significant-other support. The bootstrap intervals overlapped, and the three-way shared commonality component represented 61.6% of the incremental R^2^. The ranking is a sample-specific description of overlapping support dimensions.

Family and intergenerational ties remain prominent sources of support in later life (36, 44). Friend support also has distinct associations with social participation and loneliness (45). In this sample, family and friend support accounted for the two largest dominance weights, while significant-other support retained a positive separate-model coefficient. The overlapping intervals and commonality results place the three estimates within a shared support structure.

Later-life tourism research connects travel with healthy aging, social participation, and meaningful experience (46, 47). Wellness-tourism research also reports associations among service experiences, restorative appraisal, life satisfaction, and well-being (20). The present facility served as a health-seeking recruitment context. The analysis measured participant psychosocial characteristics and contained no facility-environment exposure. The findings therefore characterize voluntary older users of one thermal service setting.

The results identify two concurrent psychosocial correlates of life engagement: perceived support and aging anxiety. Related work documents social support within psychological-strain models (35) and emotional-symptom interventions among community-dwelling older adults (38). Family-support research also demonstrates strong cultural and contextual variation (48). Assessment studies in thermal-service populations can incorporate both support structure and aging-related concern, with longitudinal follow-up used to establish temporal order.

### Limitations

The study used established instruments, an adjusted interaction model, complete covariate reporting, HC3 inference, conditional slopes, and prespecified sensitivity analyses. Its cross-sectional design establishes no temporal or causal direction. All primary variables were self-reported, and common-method variance remains possible. Recruitment at one thermal facility produced a self-selected group of health-seeking older adults; generalizability to the wider older population and other facilities is limited. The invitation denominator was unavailable. Thirty-one records were excluded from the primary model, and exclusion was associated with several categorical characteristics. The strict complete-item interaction confidence interval included zero. The aging-anxiety total was treated as an operational composite without a new factor-analytic validation. The high MSPSS reliability and source correlations indicate substantial item and subscale overlap. Dominance and commonality results are sample-specific variance decompositions without causal or clinical ranking.

## Conclusion

Among older adults voluntarily using one thermal health tourism facility, perceived social support was positively associated with life engagement and aging anxiety was negatively associated with life engagement. The positive support-life engagement association was smaller at higher aging-anxiety levels, with a modest increment in explained variance. Sensitivity analyses produced mixed evidence for the interaction. Significant-other, family, and friend support contributed mainly through shared variance. These findings define a cross-sectional psychosocial pattern for this recruitment context.

## Data Availability

The original contributions presented in the study are included in the article/Supplementary Material, further inquiries can be directed to the corresponding author.
